# Tracking daratumumab clearance using mass spectrometry: implications on M protein monitoring and reusing daratumumab

**DOI:** 10.1038/s41375-021-01501-0

**Published:** 2022-01-29

**Authors:** Nadine Abdallah, David Murray, Angela Dispenzieri, Prashant Kapoor, Morie A. Gertz, Martha Q. Lacy, Suzanne R. Hayman, Francis K. Buadi, Wilson Gonsalves, Eli Muchtar, Nelson Leung, David Dingli, Taxiarchis Kourelis, Rahma Warsame, Moritz Binder, Robert A. Kyle, S. Vincent Rajkumar, Shaji Kumar

**Affiliations:** 1 Division of Hematology, Mayo Clinic, Rochester, MN USA; 2 Department of Laboratory Medicine and Pathology, Rochester, MN USA; 3 Division of Nephrology, Mayo Clinic, Rochester, MN USA

**Keywords:** Myeloma, Targeted therapies

## To the Editor:

The increased use of daratumumab, a CD38 targeted IgG1κ monoclonal antibody [1], in the treatment of multiple myeloma (MM) and related plasma cell disorders has introduced challenges in the accurate assessment of treatment response with gel-based techniques due to difficulty distinguishing therapeutic monoclonal antibodies and endogenous disease-related monoclonal (M) proteins sharing the same migration pattern and isotype [2]. Immunoglobulin enrichment coupled with matrix-assisted laser desorption ionization time-of-flight mass spectrometry termed MASS-FIX is a mass spectrometry-based assay with superior sensitivity in detecting M proteins over conventional gel-based techniques [3, 4], and ability to differentiate therapeutic monoclonal antibodies from endogenous M proteins based on unique retention time and mass [5, 6], replacing immunofixation in routine practice at Mayo Clinic since 2018. As the utilization of monoclonal antibodies increases, it becomes increasingly important to understand their persistence patterns in the serum of treated patients; mass spectrometry-based assays provide an ideal opportunity for this evaluation. This study was designed to evaluate the duration of daratumumab detection by MASS-FIX in the serum of treated patients and identify factors associated with clearance.

Using a prospectively maintained database, we identified 118 patients who received daratumumab for the treatment of a plasma cell disorder anytime during their disease course between March 15, 2016, and July 31, 2020, and who had at least one MASS-FIX result available within 12 months of daratumumab discontinuation. The method for MASS-FIX testing has been previously described [7]. We then collected serial MASS-FIX data obtained after daratumumab discontinuation until last follow-up, and evaluated the association between daratumumab clearance and clinical/laboratory parameters from the time of discontinuation. We measured the time to daratumumab clearance defined as time from the last daratumumab dose to the first MASS-FIX test on which daratumumab was not detected, with the observation censored at the last MASS-FIX date if still detected. We also assessed factors associated with the time to clearance using univariate Cox proportional hazards models; odds ratios (OR) for daratumumab clearance were calculated. Hypogammaglobulinemia was defined as IgG level below the lower limit of normal based on our laboratory reference range (767–1590 mg/dl). The time to clearance was plotted using the Kaplan–Meier method and compared between groups using the Log-rank test. Two-sided *p* values <0.05 were considered statistically significant. Statistical analysis was performed using JMP® software, SAS Institute Inc., Cary, NC. The study was approved by Mayo Clinic Institutional Review Board and informed consent was available for all included patients.

The median age of included patients (118 patients) was 65 years, and the most common diagnoses were MM (72%) and light chain amyloidosis (19%). Daratumumab-based treatments were initiated after 26.0 [interquartile range (IQR):6.1–71.5] months from initial diagnosis. The median duration of daratumumab-based treatment was 6.7 (IQR: 3.0–15.5) months. Table 1 shows patient characteristics including diagnosis, treatment regimen, and parameters at the time of daratumumab discontinuation. The median follow-up with serial MASS-FIX from the time of daratumumab discontinuation was 15.0 (95% CI: 9.7–not reached) months. By last follow-up, daratumumab was not detected by MASS-FIX in 77% of patients but remained detectable in 23%. The median time to disappearance by MASS-FIX was 6.1 (95% CI: 4.6–6.7) months. Among patients where daratumumab was undetectable by last follow-up, the median time from daratumumab discontinuation to disappearance was 5.1 (IQR: 3.7–7.1) months; the range was 0.5–17.0 months. When analysis was restricted to patients who had their last MASS-FIX test within 3 months of the prior positive one obtained after daratumumab discontinuation (53 patients), the median time from daratumumab discontinuation to disappearance was 4.3 (95% CI: 3.5–5.6) months. Among patients where daratumumab was undetectable, the median time to disappearance was 4.3 (IQR: 3.1–6.7) months; the range was 0.5–12.9 months.

**Table 1:** Baseline patient characteristics (*N* = 118).

	*N* (%)	Median (IQR)
Age at daratumumab discontinuation (years)		65 (58–70)
Age ≥70 years at daratumumab discontinuation	33 (28)	
Serum heavy chain isotype (*N* = 94)		
IgG	41 (44)	
IgA	23 (24)	
None	26 (28)	
Indeterminate	4 (4)	
Serum light chain isotype (*N* = 94)		
Kappa	45 (48)	
Lambda	35 (37)	
None	10 (11)	
Indeterminate	4 (4)	
Diagnosis (*N* = 118)		
Multiple myeloma	85 (72)	
Light chain amyloidosis	22 (19)	
Osteosclerotic myeloma/POEMS	3 (3)	
Monoclonal gammopathy of renal significance	5 (4)	
Monoclonal gammopathy of undetermined significance with AIHA	1 (<1)	
Cryoglobulinemia	1 (<1)	
Plasma cell leukemia	1 (<1)	
Daratumumab-containing regimen (*N* = 118)		
DVd	26 (22)	
Dara or Dd	25 (21)	
DPd	22 (19)	
DRd	22 (19)	
D-IRd	12 (10)	
DKd	3 (3)	
D-CYBORD	2 (2)	
DCd	2 (2)	
D-KRd	1 (<1)	
D-KPd	1 (<1)	
Dara-DTPACE	1 (<1)	
D-VTP	1 (<1)	
Daratumumab schedule at discontinuation (*N* = 100)		
Q 1 or 2 weeks	52 (52)	
Q >2 weeks	48 (48)	
IV/SQ (*N* = 113)	112/1	
ASCT after daratumumab-based induction	32 (27%)	
Data at the time of daratumumab discontinuation		
Creatinine (mg/dl) (*N* = 85)		1.03 (0.87–1.43)
eGFR <60 (ml/min/1.73 m^2^) (*N* = 78)	31 (40)	
Urine protein >0.5 (grams/24 h) (*N* = 65)	17 (26)	
ALKP (IU/l) (*N* = 94)		71 (58–87)
Bilirubin (mg/dl) (*N* = 63)		0.4 (0.3–0.5)
Weight (kg) (*N* = 78)		85.1 (73.4–93.2)
BMI ≥ 30 (*N* = 78)	25 (32%)	
Immunoglobulins (mg/dl)		
IgG (*N* = 85)		323 (239–464)
IgA (*N* = 78)		23 (11–83)
IgM (*N* = 62)		14 (8–25)
Hypogammaglobulinemia (*N* = 85)	80 (94)	

*AIHA* autoimmune hemolytic anemia, *ASCT* autologous stem cell transplantation, *BMI* body mass index, *Dara-DTPACE* daratumumab + dexamethasone + thalidomide + cisplatin + doxorubicin + etoposide, *DCd* daratumumab + cyclophosphamide + dexamethasone, *D-CYBORD* daratumumab + cyclophosphamide + bortezomib + dexamethasone, *Dd* daratumumab + dexamethasone, *D-IRD* daratumumab + Ixazomib + lenalidomide + dexamethasone, *DKd* daratumumab + carfilzomib + dexamethasone, *D-KPd* daratumumab + carfilzomib + pomalidomide + dexamethasone, *D-KRd* daratumumab + carfilzomib + lenalidomide + dexamethasone, *DPd* daratumumab + pomalidomide + dexamethasone, *DRd* daratumumab + lenalidomide + dexamethasone, *DVd* daratumumab + bortezomib + dexamethasone, *D-VTd* daratumumab + bortezomib + thalidomide + dexamethasone, *IV* intravenous, *IQR* interquartile range, *SQ* subcutaneous.

On univariate analysis, proteinuria (≥0.5 grams/24 h) was associated with earlier disappearance of daratumumab (OR: 2.79, 95% CI: 1.39–5.62), with median time of 3.8 (95% CI: 2.5–5.7) and 6.2 (95% CI: 4.6–7.1) months in patients with urine protein ≥0.5 and <0.5 grams/24 h, respectively (*p* = 0.003). Albuminuria (≥0.5 grams/24 h) was also associated with earlier disappearance of daratumumab (OR: 3.00, 95% CI: 1.36–6.61, *p* = 0.007). Obesity (BMI ≥ 30) was associated with longer time to disappearance (OR: 0.60, 95% CI: 0.35–1.03), with median time of 7.1 (95% CI: 4.4–10.4) and 5.8 (95% CI: 4.3–6.3) months in patients with BMI ≥ 30 and <30, respectively, but this did not reach statistical significance (*p* = 0.06). There was no association between time to disappearance of daratumumab and age ≥70 years (OR: 0.73, 95% CI: 0.46–1.16, *p* = 0.18), male gender (OR: 0.72, 95% CI: 0.48–1.10, *p* = 0.13), creatinine ≥2 mg/dl (OR: 0.86, 95% CI: 0.37–2.01, *p* = 0.73), eGFR <60 ml/min/1.73 m^2^ (OR: 1.21, 95% CI: 0.72–2.04, *p* = 0.47), albumin <3.5 g/dl (OR: 1.14, 95% CI: 0.68–1.92, *p* = 0.62), daratumumab schedule (every 1/2 weeks vs. >2 weeks) (OR: 1.27, 95% CI: 0.81–2.00, *p* = 0.30), duration of daratumumab use (<24 weeks vs. ≥24 weeks) (OR: 0.68, 95% CI: 0.44–1.04, *p* = 0.08), or hypogammaglobulinemia (OR: 2.79, 95% CI: 0.39–20.21, *p* = 0.31), even when an IgG cutoff of 500 mg/dl was used (OR: 0.67, *p* = 0.22). Among MM patients, there was no association between daratumumab disappearance by MASS-FIX and IgG (vs. IgA) isotype (OR: 0.89, 95% CI: 0.45–1.73, *p* = 0.72).

Daratumumab exhibits target-mediated drug disposition reflected by non-linear pharmacokinetics [8], with intracellular catabolism following pinocytosis or receptor-mediated endocytosis being the major elimination pathway; the neonatal Fc receptor (FcRn) also plays a role in daratumumab disposition by protecting it from catabolism and extending its half-life [9, 10]. Despite widespread use, its persistence pattern in the serum after discontinuation has not been well studied. In the SIRIUS study, daratumumab concentration measured at 8 weeks from the last infusion had decreased below quantifiable levels with the 8 mg/kg dosing but was still detectable in most patients with the 16 mg/kg dosing [11]. A previous retrospective study evaluating detection patterns of elotuzumab and daratumumab by gel-based techniques in 16 individual treatment courses showed elotuzumab persistence for 70 days after discontinuation and variability in daratumumab detection during the maintenance phase, with persistent detection up to 11 days from discontinuation in the 2 patients who had discontinued daratumumab during the study period [2]. Our study, including 118 patients and based on MASS-FIX, shows that daratumumab remains detectable for several months after discontinuation. Although the frequency, interval, and duration of serial MASS-FIX testing was variable among patients, all major limitations of our analysis, we observed persistence after treatment discontinuation exceeding 12 months in some patients, even when analysis was restricted to patients who had their last MASS-FIX test within 3 months of the prior positive test. Furthermore, we observed wide variation in the duration of daratumumab detection, reflecting the influence of patient, disease and/or treatment-related factors on daratumumab clearance. Among the factors evaluated in this study, proteinuria was associated with earlier disappearance of daratumumab by MASS-FIX. Although renal excretion plays limited role in elimination of monoclonal antibodies due to their large molecular size, glomerular injury resulting from various disease conditions including light chain amyloidosis may lead to increased renal losses and thus increased clearance of these antibodies [10]. We also observed a non-statistically significant trend toward delayed disappearance in patients with obesity; although increasing body weight is associated with increased volume of distribution and clearance, which is the basis of weight-based dosing [12], the exact relationship between obesity and daratumumab disposition is not completely understood, and is likely influenced by several factors with opposing pharmacokinetic effects including blood volume, lymphatic drainage, inflammatory biomarkers affecting catabolism, role of the FcRn, and impact of associated comorbidities, among other factors [13]. We did not observe an association between daratumumab disappearance and hypogammaglobulinemia or non-IgG isotype, although both factors are theoretically expected to result in decreased clearance due to decreased competition for FcRn [12, 14]. This may be due to small sample size, where >90% had hypogammaglobulinemia, and/or reflects a relatively small contribution of FcRn in daratumumab clearance. We were unable to assess the impact of the route of daratumumab administration on daratumumab clearance by MASS-FIX as almost all included patients received intravenous daratumumab.

Despite limitations of this study, our results showing long-term persistence of daratumumab after discontinuation have important implications on diagnostic testing and disease monitoring in both clinical trials and practice, where interference by therapeutic monoclonal antibodies confounds accurate complete response assessments with gel-based techniques. In addition, these results have implications on reuse of daratumumab in subsequent treatment lines. Larger longitudinal studies with uniform follow-up at regular intervals are needed to accurately determine the range of persistence for daratumumab and other therapeutic monoclonal antibodies and evaluate additional factors associated with clearance.
