# Correction: Tracking daratumumab clearance using mass spectrometry: implications on M protein monitoring and reusing daratumumab

**DOI:** 10.1038/s41375-022-01567-4

**Published:** 2022-04-12

**Authors:** Nadine Abdallah, David Murray, Angela Dispenzieri, Prashant Kapoor, Morie A. Gertz, Martha Q. Lacy, Suzanne R. Hayman, Francis K. Buadi, Wilson Gonsalves, Eli Muchtar, Nelson Leung, David Dingli, Taxiarchis Kourelis, Rahma Warsame, Moritz Binder, Robert A. Kyle, S. Vincent Rajkumar, Shaji Kumar

**Affiliations:** 1 Division of Hematology, Mayo Clinic, Rochester, MN USA; 2 Department of Laboratory Medicine and Pathology, Rochester, MN USA; 3 Division of Nephrology, Mayo Clinic, Rochester, MN USA

**Keywords:** Myeloma, Targeted therapies

Correction to: *Leukemia* 10.1038/s41375-021-01501-0, published online 29 January 2022

We noted a mistake in the Introduction section in the following sentence: “and ability to differentiate therapeutic monoclonal antibodies from endogenous M proteins based on unique retention time and mass”. The use of mass and retention time is actually not true for the MALDI method. We would like to correct this sentence and replace it with: “with ability to aid in differentiating therapeutic monoclonal antibodies from endogenous M proteins on the basis of mass to charge”.

