# Correction: Tracking daratumumab clearance using mass spectrometry: implications on M protein monitoring and reusing daratumumab

**DOI:** 10.1038/s41375-024-02308-5

**Published:** 2024-06-17

**Authors:** Nadine Abdallah, David Murray, Angela Dispenzieri, Prashant Kapoor, Morie A. Gertz, Martha Q. Lacy, Suzanne R. Hayman, Francis K. Buadi, Wilson Gonsalves, Eli Muchtar, Nelson Leung, David Dingli, Taxiarchis Kourelis, Rahma Warsame, Moritz Binder, Robert A. Kyle, S. Vincent Rajkumar, Shaji Kumar

**Affiliations:** 1 Division of Hematology, Mayo Clinic, Rochester, MN USA; 2 Department of Laboratory, Medicine and Pathology, Rochester, MN USA; 3 Division of Nephrology, Mayo Clinic, Rochester, MN USA

**Keywords:** Myeloma, Targeted therapies

Correction to: *Leukemia* 10.1038/s41375-021-01501-0, published online 29 January 2022

The Funding information section was missing from this article and should have read ‘This work was supported by the Paul Calabresi K12 Career Development Award (CA90628-21).’.

The original article has been corrected.

